# Effect of Marital Self-Disclosure Intervention on Fear of Cancer Recurrence and Dyadic Coping Ability Among Gastric Cancer Patients and Their Spouses: A Quasi-Experimental Study

**DOI:** 10.3390/healthcare14172875

**Published:** 2026-09-07

**Authors:** Haiyan Zhao, Ye Zhou, Chunyan Wu, Yuzhu Hou, Ying Ji, Yun Liu, Hejuan Cheng

**Affiliations:** 1 Department of Nursing, Jingjiang People’s Hospital Affiliated to Yangzhou University, Taizhou 214500, China; air000001@126.com (H.Z.); 13914500132@163.com (C.W.); frankerji3333@163.com (Y.H.); ji18052621938_ji@163.com (Y.J.);; 2 Department of Nursing Science, Faculty of Medicine, Universiti Malaya, Kuala Lumpur 500088, Malaysia; yeyecrystal@126.com; 3 Editorial Office of International Journal of Nursing Sciences, Chinese Nursing Journals Publishing House Co., Ltd., Beijing 100035, China

**Keywords:** couple, marital self-disclosure, gastric cancer, quasi-experimental study, spouse

## Abstract

Background: Fear of cancer recurrence (FCR) may extend beyond the individual patient, affecting spouses and the couple’s psychological adjustment and dyadic coping. Therefore, interventions that address both patients and their spouses and promote effective communication and dyadic coping may offer a promising approach to alleviating FCR and improving psychological well-being. Objective: This research seeks to evaluate how a marital self-disclosure intervention influences FCR and enhances dyadic coping in gastric cancer patients and their partners. Methods: We undertook a quasi-experimental study from October 2022 to April 2024. Participants in the experimental group completed 4 phases of the marital self-disclosure (MSD) intervention after disclosure guidance; the intervention included verbal and written self-disclosure. Participants in the control group received usual psychological care. We evaluated patients at baseline, 8 weeks, and 16 weeks. The primary outcome was the FoP-Q-SF score for patients. Secondary outcomes were the FoP-Q-SF scores for partners and the Dyadic Coping Inventory (DCI). Results: The experimental and control groups each included 42 patients, with 38 and 36 participants completing the 16-week follow-up, respectively. The results showed statistically significant inter-group, time, and interaction effects of FoP-Q-SF scores for Patients and Partners (*p* < 0.05). The inter-group, time, and interaction effects of the DCI on the scores of the five dimensions and overall were all statistically significant (*p* < 0.05). Conclusions: It is essential to reduce FCR and enhance dyadic coping ability among individuals with gastric cancer. This research demonstrated that the MSD intervention was more effective than routine nursing care in alleviating FCR.

## 1. Introduction

Gastric cancer is the fifth most commonly diagnosed malignancy worldwide and the fourth leading cause of cancer-related mortality. In 2020, approximately 1.09 million new cases and 769,000 deaths were reported globally, representing 5.6% of all cancer diagnoses and 7.7% of cancer deaths [1]. The age-standardized incidence and mortality rates were 11.1 and 7.7 per 100,000, respectively, with lifetime risks (ages 0–74) of 1.31% for developing and 0.90% for dying from this disease. Recent data from the National Cancer Registry indicate that, in China, gastric cancer is the second most frequent cancer in both incidence and mortality, following lung cancer. In 2020, China recorded about 679,000 new cases and 498,000 deaths, while the disease contributed 9.83 million disability-adjusted life years (DALYs), accounting for over 44% of the global burden [2]. Advances in adjuvant chemotherapy have significantly improved survival outcomes in gastric cancer, with long-term survival benefits demonstrated in patients receiving fluoropyrimidine- and oxaliplatin-based regimens [3]. Nevertheless, despite surgical resection and combined drug therapy prolonging survival, the high likelihood of recurrence and metastasis continues to impose substantial psychological distress on patients.

An international consensus panel of 65 psycho-oncology experts defined severe fear of cancer recurrence (FCR) as a persistent condition, lasting at least three months, characterized by excessive concern, intense preoccupation, and heightened vigilance toward physical sensations [4]. Empirical studies on gastric cancer patients, typically involving 120–170 participants, reported FCR scores (FOP-Q-SF) ranging from 35.43 to 42.30, reflecting moderate-to-high levels. Within its subdimensions, physical health scored 18.09 ± 4.52, while the social/family dimension scored 17.34 ± 6.34 [5,6]. Compared with breast cancer [7], gastric cancer patients showed higher overall FCR, with greater concern for physical health than for family or social aspects. The disease’s characteristics—such as hidden onset, frequent misdiagnosis, high metastatic potential, and elevated recurrence rate—exacerbate patients’ fears [1]. Adjuvant chemotherapy after surgery remains the cornerstone of treatment and improves survival. However, adverse effects, including nausea, vomiting, mucosal dryness, constipation, and bone marrow suppression, not only diminish physical well-being but also amplify the anticipation of relapse and progression. Moreover, the extended treatment cycles and prolonged nutritional dependence place patients in homebound states, reduce household income, and simultaneously increase medical costs. This dual economic and psychological strain further aggravates FCR [8]. Sustained high levels of FCR are associated with maladaptive behaviors such as avoidance, hypersensitivity to bodily changes, difficulty in planning, and declining quality of life. These consequences can persist for years beyond treatment, and in severe cases, may culminate in anxiety disorders, post-traumatic stress symptoms, or depressive episodes. Despite mounting evidence linking FCR to poor mental health outcomes and reduced adherence to treatment, it is still underestimated or overlooked in routine oncology care.

Emotional self-disclosure can enhance relationship intimacy, which may, in turn, help reduce FCR. Emotional disclosure is the genuine communication of one’s inner thoughts and feelings, whether spoken or written. It serves as an avenue for individuals to share emotions, ideas, and perspectives with their partners. Conceptually, it has been described in two dimensions: (a) the extent to which individuals openly express their thoughts and emotions, and (b) the extent to which they inhibit or withhold such expression. Within marital relationships, emotional disclosure represents a fundamental aspect of mutual emotional support, encompassing expressions of care, love, and empathy. For married or partnered individuals coping with cancer, the spousal relationship plays a pivotal role in their psychological adaptation to illness. Zhou et al. [9] highlighted that cancer-related stressors can put couples at risk of relational strain. A significant contributor to this strain is the difficulty many couples face in discussing cancer-related issues openly. While cancer patients frequently identify their spouse as their primary confidant, many still experience hesitation in sharing their worries. At the same time, partners may withdraw or distance themselves from the patient’s distress, even in relationships that are generally perceived as satisfying [10]. When patients are unable to express concerns freely, both their psychological adjustment and the overall quality of the marital relationship may be undermined. Conversely, open dialogue about cancer-related challenges fosters better mutual understanding, enabling partners to provide more appropriate and effective support. This exchange benefits not only patients but also partners. Evidence suggests that partner distress intensifies when patients silently carry their worries, whereas partners may feel relief when patients disclose their fears openly, reducing partner depression [10].

Multiple linear regression analysis has indicated that both self-disclosure and marital intimacy are significant predictors of FCR among patients [11]. However, most intervention studies to date have primarily emphasized the effect of self-disclosure on patients’ psychological well-being, often overlooking the spouse’s role in shaping the patient’s emotional state. Furthermore, few studies have approached the patient and partner as a dyadic unit. Given the substantial differences between Western and Chinese contexts—including ethnicity, culture, religion, and socioeconomic development—theoretical frameworks, assessment tools, and analyses of influencing factors derived from Western populations may not directly apply to Chinese patients. For individuals with advanced gastric cancer, the prolonged treatment duration and elevated recurrence risk add considerable psychological stress [10]. Chemotherapy brings additional challenges, including dietary restrictions and treatment-related side effects such as nausea, vomiting, reflux, and loss of appetite [12]. Evidence from studies of breast and other common cancers has largely centered on partner communication, dyadic coping, and psychosocial adaptation throughout the cancer trajectory [13]. However, these findings cannot be fully generalized to gastric cancer due to the disease’s unique clinical course and treatment burden. Current research on communication between gastric cancer patients and their spouses remains limited. Given the limited research on emotional self-disclosure between gastric cancer patients and their spouses, this study innovatively developed a culturally tailored marital self-disclosure (MSD) intervention for Chinese patients and their partners, aiming to reduce fear of cancer recurrence (FCR) and enhance dyadic coping ability (DCA) among couples during cancer chemotherapy.

## 2. Methods

### 2.1. Theoretical Framework

**Figure 1:**
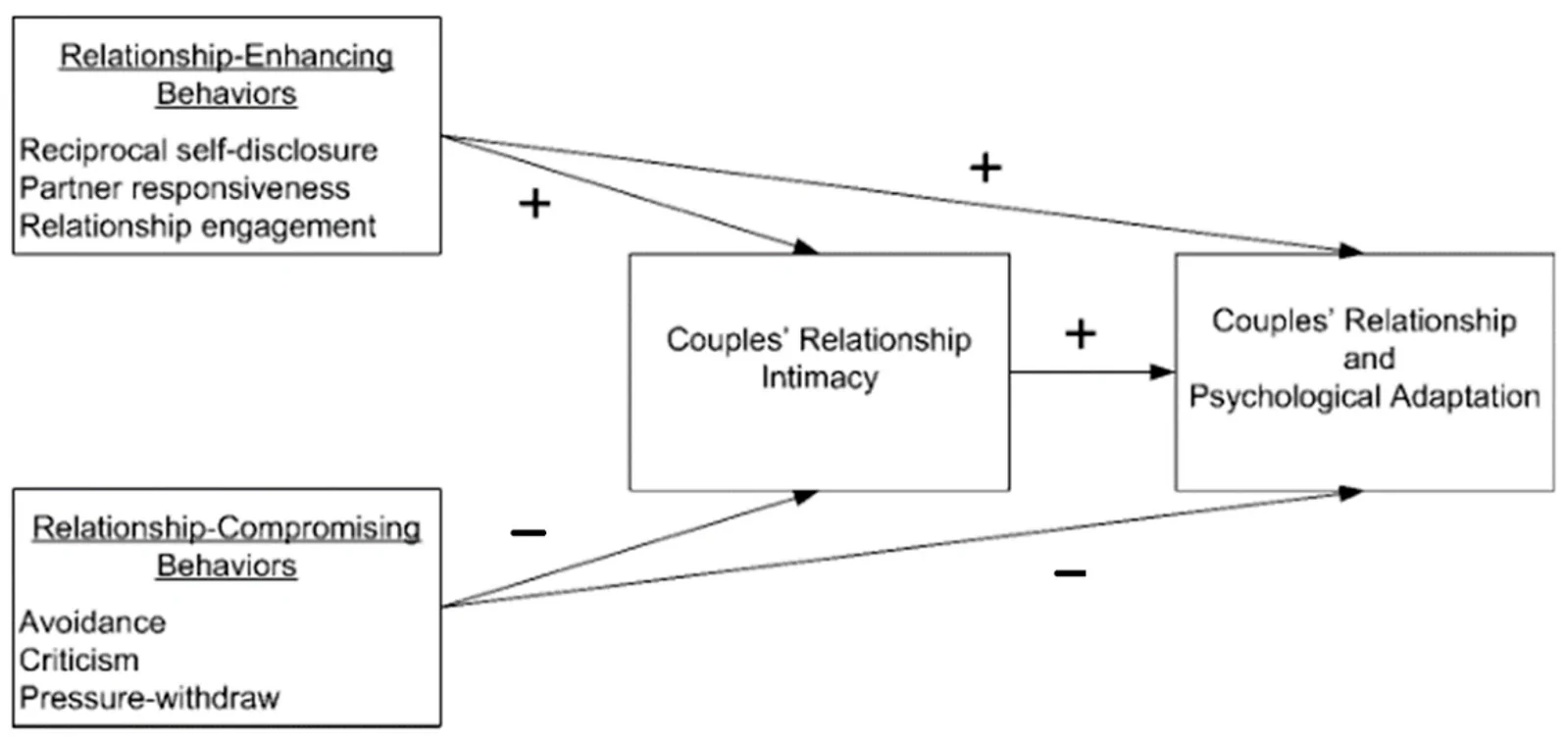
The Relationship Intimacy Model of Couple Adaptation to Cancer. Note: “+” is positive effect; “−” is negative effect.

Manne and Badr [14] argued that it is essential to assess not only cancer patients’ psychological adaptation but also that of their partners. Building on theories such as resource theory and social cognition theory, they introduced a model that examines the intimacy dynamics between couples coping with cancer, as shown in Figure 1. This model identifies two main categories of behaviors that affect psychological adjustment: relationship-enhancing and relationship-compromising. Relationship-enhancing behaviors—such as reciprocal self-disclosure, partner responsiveness, and relationship engagement—are positively associated with both intimacy and psychological adaptation. On the other hand, relationship-compromising behaviors—such as avoidance, criticism, and pressure withdrawal—diminish intimacy, relationship quality, and psychological adaptation. Manne et al. [15] particularly emphasized that reciprocal self-disclosure is a key factor in relationship-enhancing behaviors that supports the psychological adaptation of both cancer patients and their partners, especially in the face of cancer-related challenges. This model serves as a theoretical framework for the current study, which explores how mutual self-disclosure influences couples’ relationship intimacy and their overall psychological adaptation.

Based on the Couples’ Coping with Cancer Intimacy Model [15], this study focuses on reciprocal self-disclosure within relationship-enhancing behaviors as a key intervention. The goal is to promote intimacy in couples’ relationships and improve their psychological adaptation. The MSD designed in this study aimed to reduce distress among couples and increase their relationship intimacy through a chemotherapy/hospitalisation intervention or chemotherapy interval/home intervention, thereby reducing FCR.

### 2.2. Study Design

This study explores the effect of MSD on FCR and dyadic coping ability in patients with gastric cancer and their spouses. This quasi-experimental study used a nonequivalent (pretest-posttest) control group design. Our team conducted it from October 2022 to April 2024.

### 2.3. Setting

We recruited participants from these two hospitals. The two hospitals are in the same city and have similar characteristics. To prevent contamination, we randomly assigned participants: those from Hospital A were allocated to the experimental group, and those from Hospital B were allocated to the control group. The treatment methods followed the “Guidelines for Diagnosis and Treatment of Gastric Cancer” [16]; the chemotherapy regimen was selected based on patients’ actual conditions. Chemotherapy generally lasts 4–6 cycles. After chemotherapy, patients generally enter the follow-up stage or a treatment adjustment stage. The hospital stay for each chemotherapy cycle is 3–4 days.

### 2.4. Participants

Eligible patients were adults with advanced gastric cancer confirmed by preoperative gastroscopy and pathology, currently undergoing chemotherapy, married and living with their spouse, cognitively and linguistically capable, and willing to participate. Spouses were required to cohabit, serve as the primary caregiver for at least four hours daily, be cognitively and linguistically capable, and consent to participate. Patients or spouses were excluded if they had a prior cancer diagnosis, severe complications, cognitive or psychiatric disorders, significant sensory or speech impairments, had received psychotherapy or MSD training, were involved in other research, or declined joint participation.

### 2.5. Sample Size

The primary dependent variable in this study was FCR, measured using the FoP-Q-SF. The sample size for this study was calculated based on the FoP-Q-SF scores from the referenced literature [17]. Sample size = 2 (SD) 2 × (Zα/2 + Zβ)2 ÷ D2 = 2 (4.25) 2 × (1.96 + 0.84)2 ÷ 2.882 = 36.125 × 7.84/8.2944 = 34.14 = 35.

The sample size for each dependent variable was *n* = 35 for FCR. To address dropout, we added an additional 20% to the calculation. As a result, the sample sizes for the experimental and control groups are 42 each. Based on the calculation formula, accounting for attrition rates, a sample size of 42 cases per group is deemed sufficient to test this study’s hypotheses.

### 2.6. Human Ethics and Consent to Participate

The study received approval from the Ethics Committees of the two participating hospitals (Approval Nos. TZRY-LL-AF/SQ-018-3.0, KY 2022-167-01) and was conducted in accordance with the Declaration of Helsinki. It was registered on ClinicalTrials.gov in November 2022 (NCT05606549). We obtained written informed consent from all participants before enrollment. The study strictly followed ethical standards to protect participants’ rights, including autonomy, privacy, anonymity, confidentiality, and equitable treatment, as well as to safeguard them from potential discomfort or harm.

### 2.7. Recruitment, Randomization, and Blinding

Clinical research nurses (Ying Ji and Yun Liu) recruited eligible participants (patients with gastric cancer and their spouses) from the oncology departments of 2 tertiary hospitals in Taizhou City, Jiangsu province. Oncology nurse practitioners informed eligible patients about the study when they were first diagnosed with gastric cancer. If the patient and their spouse were interested, the oncology nurse practitioner immediately informed the researcher. The researcher then introduced the study’s purpose, benefits, and procedures to patients with gastric cancer and their spouses and assured participants that their details would be protected; participation was voluntary, and they could withdraw at any time without affecting their therapy or nursing. The data were kept anonymous and confidential. We obtained informed consent from those who agreed to participate.

To avoid contamination, we randomly selected two hospitals. Two data collectors (one in the experimental group and one in the control group) received the same standardized training in data collection. They were not involved in recruitment, intervention implementation, or data analysis, but they collected the outcomes (scores on the FoP-Q-SF and the Dyadic Coping Inventory). Throughout the study, the intervention nurses, patients, and their spouses were aware of group assignments, making blinding unfeasible.

### 2.8. Control Group (Routine Nursing Care)

The control group received routine nursing care. Routine nursing care refers to the “practical nursing of oncology” [18]. It includes routine nursing related to the patient’s treatment, observation of chemotherapy-related toxic and side effects, guidance on the use of therapeutic drugs, exercise and nutrition management, and psychological support and life guidance during the chemotherapy interval. Upon hospital admission, patients receive standard nursing care related to their treatment, along with a booklet covering gastric cancer and treatment information, chemotherapy side effects, nutrition and exercise guidance, and psychological support (e.g., relaxation techniques, music), as well as daily-life guidance during chemotherapy intervals. However, nurses do not receive specific training in communication skills with patients and generally do not actively encourage couples to discuss their cancer-related thoughts or emotions.

### 2.9. Intervention of the Experimental Group (MSD)

In addition to routine nursing care (identical to that of the control group), the experimental group received the MSD intervention. The MSD intervention program was developed through a combination of literature review, stakeholder interviews, and Delphi expert consultation. Our team has described the program’s detailed content in our published papers [19].

#### 2.9.1. Content of MSD Intervention for Alleviating the FCR in Patients with Gastric Cancer Undergoing Chemotherapy

**Figure 2:**
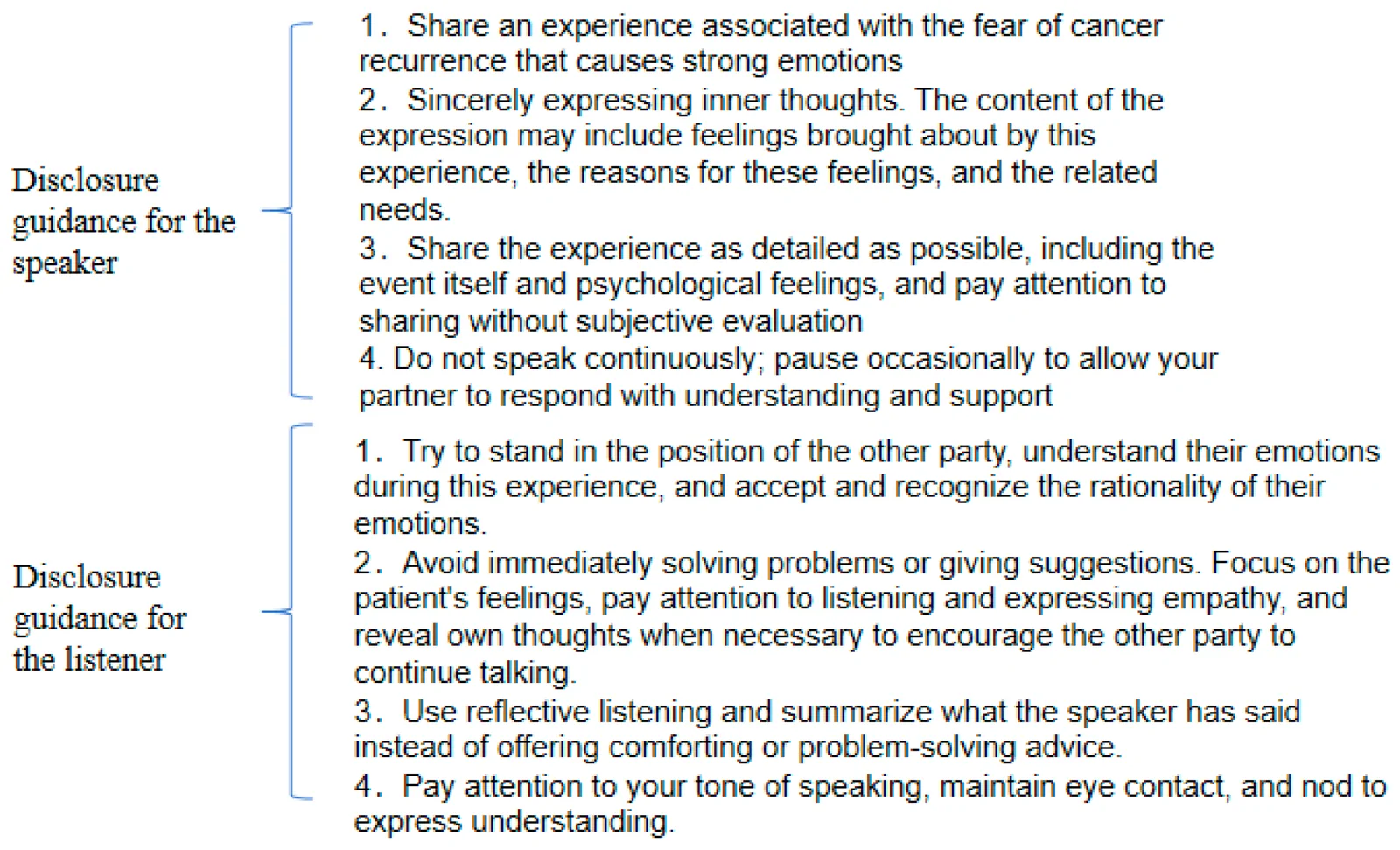
Disclosure Guidance for Patients and Their Spouses. Note: The patient and their spouse take turns being the speaker and the listener.

**Figure 3:**
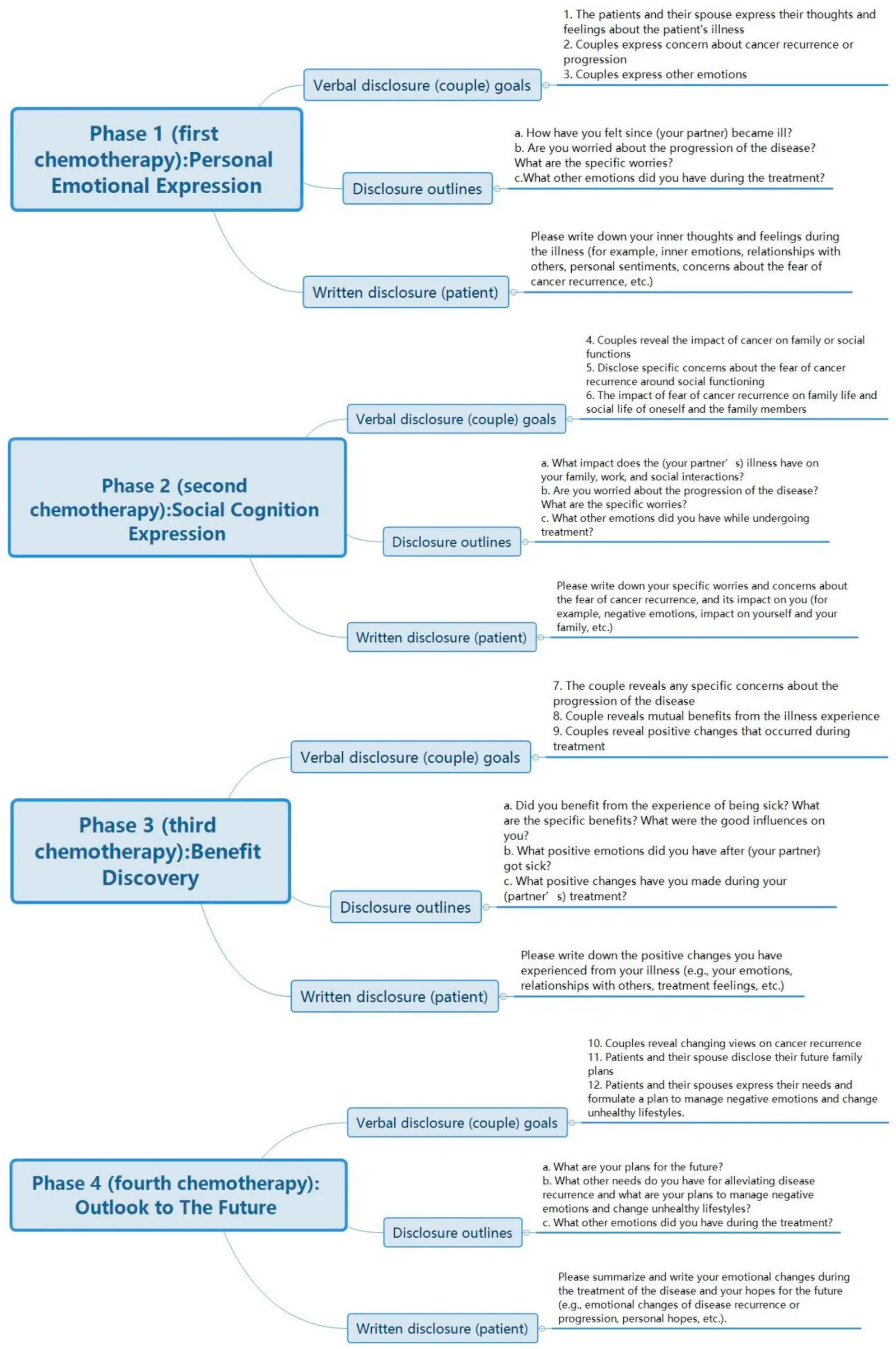
The structure and themes of MSD.

The research team conducted group discussions to finalize the disclosure program, drawing on expert consultations and the Delphi method. The content of the MSD intervention was as follows:

MSD intervention was conducted in a quiet, comfortable environment, starting with verbal disclosure and followed by written disclosure. Patients wrote about their experiences focusing on specific themes. Verbal sessions lasted 20 to 30 min each, and written sessions did as well. For hospitalized patients undergoing chemotherapy, the nurse led the MSD intervention during each chemotherapy cycle (4 cycles total). During outpatient chemotherapy intervals, MSD sessions between patients and spouses occurred weekly at home. Figure 2 shows disclosure guidance for couples. It was organized around themes: Personal Emotional Expression, Social Cognition Expression, Benefit Discovery, and Outlook to the Future. The 4 stages covered specific disclosure goals and topic outlines (see Figure 3).

#### 2.9.2. The Intervention Process of MSD

Firstly, two experienced oncology nurses (Haiyan Zhao and Yuzhu Hou) with strong nursing and communication skills built trust with patients, screened them for inclusion and exclusion criteria, obtained their consent to participate in the study, and assisted those who agreed in completing the informed consent forms. Secondly, participants in the experimental group received the MSD intervention from two communication-focused nurses with master’s degrees in nursing and certification in psychological counseling. The communication-led nurses had several responsibilities: (a) avoiding negative interactions; (b) using a checklist (which included disclosure guidance for patients and their spouses, as well as MSD themes for various stages) to remind and assist patients and their spouses with disclosure; (c) ensuring that communication adhered to the guidelines; (d) directing the content of disclosure to concentrate on the speaker’s personal experience.

(1) Chemotherapy/Hospitalization

Each session included both verbal and written disclosure. Researchers introduced MSD to gastric cancer patients and their spouses by explaining its definition, benefits, stages, and timing. Patients and their spouses alternated roles as speakers and listeners, and received five to ten minutes of training or review on MSD skills before each disclosure. Figure 2 outlines the training specifics. During disclosure, the listener was expected to allow the speaker to fully express their emotions and respond with empathy and acknowledgment of the speaker’s perspective.

Each stage had distinct disclosure goals, leading to variations in the outlines. In the Personal Emotional Expression phase, the primary goals were to share the individual’s thoughts and feelings after the illness, as well as their worries about the disease’s progression. In the Social Cognition Expression phase, the focus was on discussing how cancer affected family or social dynamics and addressing specific concerns about disease progression. During the Benefit Discovery phase, the aim was to reveal the advantages gained from the illness experience, positive emotions after the illness, and any beneficial changes during treatment. Finally, in the Outlook to the Future phase, the goals included sharing plans, expressing needs, and developing a plan to prevent cancer recurrence. The gastric cancer patient and their spouse adhered to these outlines at each stage. When one person disclosed information, the other listened attentively. Following verbal disclosures, the patient also provided written disclosure based on the outlined topics. During the written disclosure process, the researcher and spouse were expected to remain silent but respond promptly to any questions from the patient.

(2) Chemotherapy Interval at Home (Homework)

In addition to conducting MSD in the hospital, patients and their spouses also engage in MSD at home (during chemotherapy intervals). The structure and themes of MSD during chemotherapy intervals are the same as those during chemotherapy. Researchers provide patients and their spouses with an MSD booklet, which outlines themes and topics for MSD at each stage, as well as topics for patient-written disclosures. Following the booklet’s guidance, patients and their spouses conduct verbal MSD, with patients engaging in written disclosures at home. The couples decided on the timing of disclosures based on their individual circumstances. They conducted verbal and written disclosures at least once a week, each lasting 20 to 30 min.

### 2.10. Quality Control

(1) Intervention fidelity in the experimental group.

Two experienced oncology nurses (Haiyan Zhao and Yuzhu Hou) who had received professional psychological training delivered the MSD intervention. Before implementing the intervention in the experimental group, both nurses underwent standardized training provided by experienced oncology psychologists. The training covered the research objectives, interview techniques, intervention content, and follow-up procedures. After the training, both nurses demonstrated proficiency in delivering the intervention.

Both nurses received a standardized MSD intervention manual that specified the content and implementation procedures. Throughout the intervention period, both nurses adhered to the same intervention content, procedures, and standardized guidance language to ensure consistent delivery.

(2) Adherence to the MSD intervention.

Both nurses added the patients or their spouses to WeChat (vision 3.8) to facilitate real-time communication and notifications, provide weekly reminders to complete the self-disclosure activities at home, and promptly answer questions from patients or their spouses. Patients or spouses were required to photograph their completed written self-disclosure activities, upload them via WeChat, and check in to document their progress. For participants who did not use WeChat, the nurses followed up on their participation in the intervention by telephone calls. The study also documented reasons for participant dropout.

Patients in the experimental group received a booklet with instructions for completing weekly written self-disclosure activities. Nurses reviewed the written records when patients returned to the hospital for their monthly chemotherapy sessions. The research team regularly reviewed intervention records and provided feedback as needed to minimize deviations from predefined procedures.

(3) Quality control of data collection.

Both data collectors participated in face-to-face simulated data collection before the study. The training covered the designated data collection time points and the interpretation of the scale items. They were instructed to use consistent guidance language during data collection. During data collection, the questionnaires were distributed and collected on site, and each was carefully checked for completeness. Any missing or incomplete responses were promptly identified and addressed.

To reduce the potential impact of medication on patients’ psychological state, all phases of the MSD intervention and questionnaire assessments were conducted after hospital admission but before the administration of chemotherapy.

### 2.11. Outcomes

The original authors granted permission to adapt the FoP-Q-SF for Patients and Partners and the Dyadic Coping Inventory.

(1) Sociodemographic and Clinical Information

Based on the literature review, the research team designed a table of socio-demographic and clinical information. The team collected demographic information on patients with gastric cancer and their spouses, including gender, age, duration of marriage, number of children, residence, religious belief, health insurance type, medical burden, average monthly household income per capita, educational level, employment status, and current or former occupation. In addition, information on clinical variables, including history of gastric surgery, cancer stage, chemotherapy regimen, and time from cancer diagnosis (months), was collected.

(2) Fear of Progression Questionnaire–Short Form (FoP-Q-SF) for Patients

The Fear of Progression Questionnaire-Short Form (FoP-Q-SF) is a unidimensional scale derived from the original Fear of Progression Questionnaire (FoP-Q). The patient version includes 12 items, such as “Fear of disease progression,” “I feel nervous before a doctor’s exam or routine checkup,” and “Fear of pain.” The FoP-Q-SF uses a 5-point Likert scale from 1 (“never”) to 5 (“always”), with total scores ranging from 12 to 60; higher scores indicate greater fear of disease progression. Kwakkenbos et al. [20] validated the scale in Korean and Dutch, confirming its reliability and applicability for patients with cancer and chronic illnesses. Qiyun [21] adapted it into Chinese, reporting a Cronbach’s alpha of 0.886 for the total scale and 0.836 and 0.804 for its two factors, with Guttman split-half coefficients of 0.855, 0.806, and 0.828, demonstrating good internal consistency. In this study, 34 patients with gastric cancer completed the FoP-Q-SF, yielding a Cronbach’s alpha of 0.852, indicating satisfactory reliability in this population.

(3) Fear of Progression Questionnaire–Short Form (FoP-Q-SF) for Partners

The FoP-Q-SF for Partners, developed by Zimmermann et al. [22] based on the FoP-Q-SF, assesses the spouse’s fear of the patient’s disease progression and consists of 12 items., like “I feel anxious at the thought that my lover’s illness may progress,” “I feel nervous before doctor’s appointments and regular checkups,” “I’m afraid my lover will be in pain,” etc. The FoP-Q-SF for Partners uses a 5-point Likert scale (1 = never, 5 = always), with total scores ranging from 12 to 60; higher scores indicate greater fear of the spouse’s disease progression. Its Cronbach’s alpha was 0.88. Qiyun [21] translated the scale into Chinese and reported good reliability and validity, with a total Cronbach’s alpha of 0.834, a Guttman split-half coefficient of 0.818, and factor-specific alphas of 0.835 and 0.699. In this study, 36 spouses of gastric cancer patients completed the scale, yielding a Cronbach’s alpha of 0.883, demonstrating satisfactory internal consistency.

(4) Dyadic Coping Inventory (DCI)

The DCI, designed to assess dyadic coping ability, was originally developed by Bodenmann based on the system interaction model and later revised [23] and consists of 37 items. Items 1–35 are scored on a 5-point Likert scale, with negatively keyed items (7, 10, 11, 15, 22, 25, 26, 27) reverse-scored, yielding a total score range of 35–175. Subscales include Stress Communication, Supportive Dyadic Coping, Delegated Dyadic Coping, Negative Dyadic Coping, and Common Dyadic Coping, while items 36 and 37 assess satisfaction and are excluded from the total score. The DCI demonstrates strong internal consistency in multiple languages (German α = 0.91; Italian α = 0.90; French α = 0.90). Xu et al. [24] translated the DCI into Chinese and reported a Cronbach’s alpha of 0.73 for the total scale; most subscales showed acceptable reliability, while two short subscales (two items each) showed lower reliability (0.51 and 0.52), which remains reasonable. In the present study, 30 patients with gastric cancer completed the DCI, which showed good internal consistency (Cronbach’s alpha = 0.852).

### 2.12. Data Collection and Analysis

Pretest data were collected from the control and experimental groups before the first chemotherapy. Pretest data were collected as a baseline (T1) for their FCR and dyadic coping ability. Participants completed the FoP-Q-SF for patients, the FoP-Q-SF for partners, and the DCI to assess FCR and dyadic coping ability. We conducted the second and third measurements at 8 weeks (T2) and 16 weeks (T3) after the intervention.

Data were analyzed using SPSS 26.0. We present the general characteristics of both spouses of patients with gastric cancer as categorical data, expressed as frequencies and percentages. We evaluated differences in baseline demographic and clinical characteristics between the experimental and control groups using independent-samples Chi-square/Fisher’s exact tests, depending on the variable type and cell counts. Most scores for FoP-Q-SF for Patients, FoP-Q-SF for Partners, and DCI did not follow a normal distribution; therefore, we present the data as Median [IQR (Q25–Q75)]. We used a 2-sample independent *t*-test to compare two groups.

We used Little’s Missing Completely at Random (MCAR) test to determine whether the missing data were missing completely at random (MCAR) or followed a systematic pattern [25]. In this study, the test yielded a chi-square value of 7.760 with 24 degrees of freedom and a *p*-value of 0.999, indicating that the missing data were completely at random. Outcome analyses were conducted on an intention-to-treat basis, with missing values addressed using multiple imputation (five iterations). The imputation model included the outcome variables at each assessment time point, group assignment, and relevant baseline demographic and clinical characteristics. We generated five imputed datasets and analyzed each dataset separately using the prespecified statistical models. We then pooled results from the five imputed datasets to obtain the final estimates.

We used the Generalized Estimating Equations (GEE) approach, based on the Generalized Linear Model (GLM), to analyze longitudinal data with repeated measurements. GEE accounts for the within-subject correlation arising from repeated observations and provides robust parameter estimates. We specified a first-order autoregressive (AR (1)) working correlation structure for the GEE models. This structure assumes that repeated measurements closer in time are more strongly correlated than measurements taken further apart, with the correlation decreasing as the time interval increases. Given the longitudinal design of this study and the repeated assessments at prespecified time points, the AR (1) structure was considered appropriate to account for within-subject correlation over time.

We used GEE to evaluate the effects of group, time, and the group-by-time interaction on the primary and secondary outcome measures over the study duration [25]. We examined the group-by-time interaction to determine whether changes in outcome measures over time differed between the experimental and control groups. GEE was used to analyze the effectiveness of MSD in alleviating FCR among patients with gastric cancer and their spouses, as well as improving dyadic coping ability among them. Inter-group comparisons were analyzed using the Mann-Whitney U test.

## 3. Results

### 3.1. Dropout Rates and Intervention Adherence

**Figure 4:**
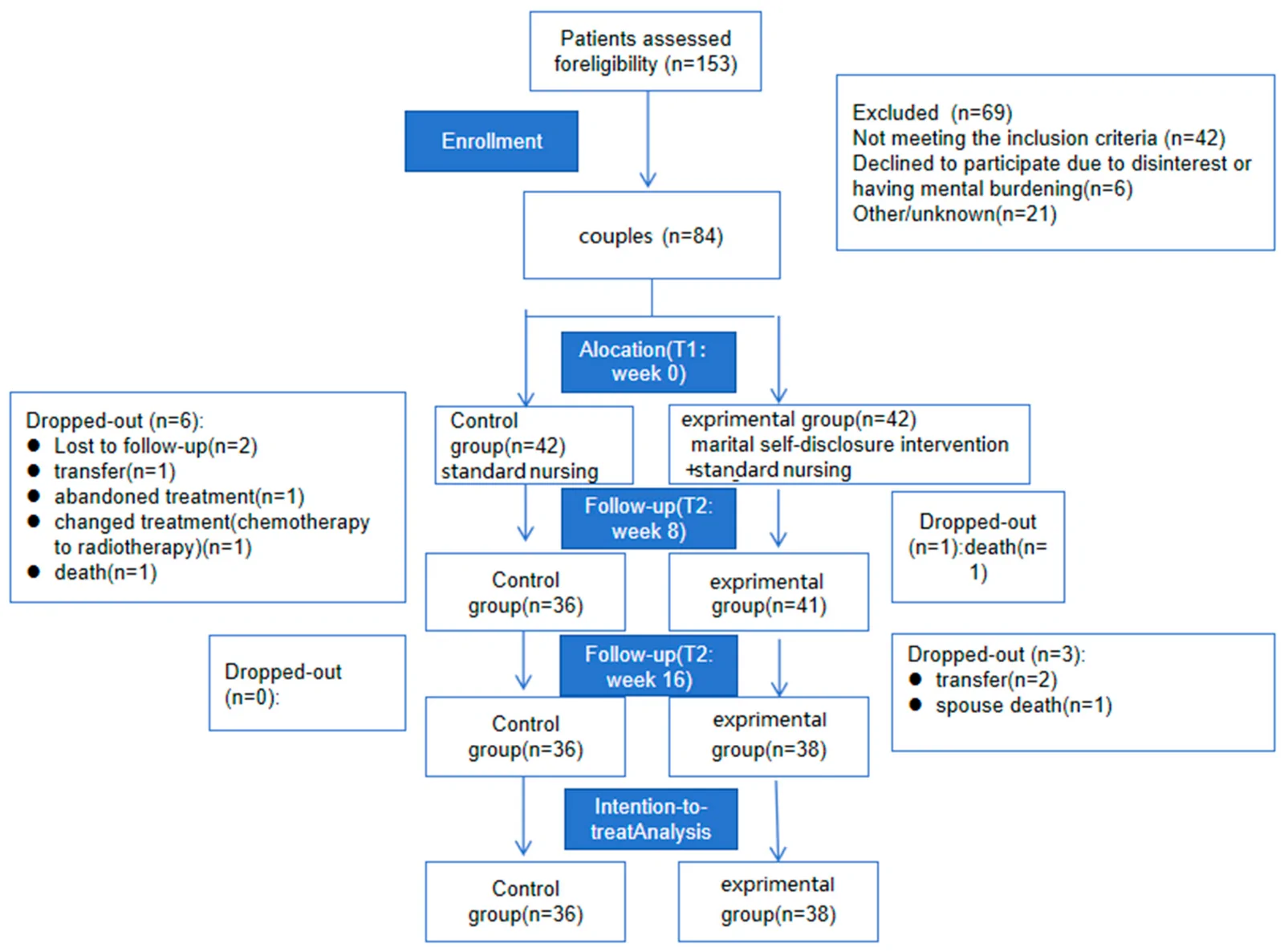
CONSORT Flow Diagram.

The study included 153 patients with gastric cancer and their spouses. A total of 84 couples met the inclusion criteria and underwent the first intervention and baseline survey (T1). In the control group, after eight weeks (T2), two couples were lost due to the inability to contact them and their family members, one couple was lost due to patient transfer to another hospital, one couple was lost due to the patient’s abandonment of treatment, one couple was lost due to a change in treatment modality (accepting radiotherapy), and one couple was lost due to patient death. Therefore, 36 couples underwent the second questionnaire survey. After 16 weeks (T3), 36 couples completed the third questionnaire. In the experimental group, after eight weeks (T2), one couple was lost due to patient death, and 41 patients underwent the second questionnaire survey. After 16 weeks (T3), one couple was lost due to the death of the spouse, and two couples were lost due to patients’ transfer to another hospital, resulting in 38 couples completing the third survey. Figure 4 shows the CONSORT flow diagram.

### 3.2. Demographic Characteristics

Table 1 and Table 2 provide demographic data for 84 couples consisting of patients with gastric cancer and their spouses, with 42 couples in both the experimental and control groups.

### 3.3. The Comparison of FoP-Q-SF for Patients and FoP-Q-SF for Partners in Two Groups (Intention-to-Treat Analysis)

A GEE analysis was conducted after multiple imputation (5 imputations) for the scores of the two groups. A Mann-Whitney U test was used for further between-group comparisons. The results of this study showed that, before the intervention, there was no statistically significant difference in scores between the FoP-Q-SF for Patients and the FoP-Q-SF for Partners (*p* > 0.05). However, GEE analysis revealed that the inter-group, time, and interaction effects of FoP-Q-SF scores for Patients and Partners were all statistically significant (*p* < 0.05). Because the group × time interaction was significant, we further analyzed the main effects. The scores of FoP-Q-SF for Patients [Group1 × T2: B = −3.532, 95%CI (−6.150, −0.914), *p* = 0.008; Group1 × T3: B = −7.311, 95%CI (−9.811, −4.811), *p* < 0.001] and for Partners [Group1 × T2: B = −3.479, 95%CI (−5.187, −1.141), *p* = 0.004; Group1 × T3: B = −5.944, 95%CI (−8.339, −3.548), *p* < 0.001] are shown in Table 3. The results showed that FoP-Q-SF scores for Patients and Partners decreased over time in both the experimental and control groups, but the decrease was greater in the experimental group at T2 and T3. Pairwise comparisons using the Mann-Whitney U test showed a similar outcome; both FoP-Q-SF scores for Patients and Partners were lower in the control group compared to the experimental group at T2 and T3, with statistically significant differences (*p* < 0.05); refer to Table 3.

### 3.4. Comparison of DCI Between Two Groups

In the experimental group, the median scores for Negative Dyadic Coping ranged from 29 to 34.32, Stress Communication ranged from 26.03 to 32, Supportive Dyadic Coping ranged from 32.05 to 40, Delegated Dyadic Coping ranged from 13.5 to 16, Common Dyadic Coping ranged from 19 to 20, and the Overall Score ranged from 119.5 to 140.29 during TI–T3. In the control group, the median scores for Negative Dyadic Coping ranged from 25.5 to 28.08, Stress Communication ranged from 25.75 to 26.64, Supportive Dyadic Coping ranged from 31.92 to 35, Delegated Dyadic Coping ranged from 13.31 to 14, Common Dyadic Coping ranged from 16.56 to 19, and the Overall Score ranged from 114.75 to 118.97 during TI–T3. Table 4 shows descriptive statistics as the median [IQR (Q25–Q75)] for the total score and each DCI dimension.

We conducted a GEE analysis after multiple imputation (5 iterations) of the two groups’ scores, followed by a Mann-Whitney U test to compare the groups. The analysis found no statistically significant differences before the intervention (T1) in Negative dyadic coping, Stress communication, Supportive dyadic coping, Delegated dyadic coping, Common dyadic coping, or Overall score (*p* > 0.05). The GEE analysis revealed that the inter-group effects, time effects, and interaction effects of the DCI for the scores of the five dimensions and the overall score were all statistically significant (*p* < 0.05), except for the interaction effects of the scores of the Common Dyadic Coping dimension, which did not show a statistically significant difference (*p* > 0.05).

Because we identified an interaction effect between group and time, we further analyzed the individual effects. Negative dyadic coping [Group1 × T2: B = 4.769, 95%CI (2.136, 7.401), *p* < 0.001; Group1 × T3: B = 5.378, 95%CI (2.776, 7.979), *p* < 0.001], Stress Communication [Group1 × T2: B = 1.607, 95%CI (−0.338, 3.552), *p* = 0.105; Group1 × T3: B = 4.215, 95%CI (2.488, 5.942), *p* < 0.001], Supportive Dyadic Coping [Group1 × T2: B = 3.435, 95%CI (1.038, 5.832), *p* = 0.005; Group1 × T3: B = 5.763, 95%CI (3.393, 8.132), *p* < 0.001], Delegated Dyadic Coping [Group1 × T2: B = 0.852, 95%CI (−0.059, 1.763), *p* = 0.067; Group1 × T3: B = 1.752, 95%CI (0.919, 2.585), *p* < 0.001], and Overall Score [Group1 × T2: B = 11.984, 95%CI (4.580, 19.387), *p* = 0.002; Group1 × T3: B = 19.402, 95%CI (12.446, 26.358), *p* < 0.01]. The results showed that scores for Negative Dyadic Coping (reverse-scored), Stress Communication, Supportive Dyadic Coping, Delegated Dyadic Coping, Common Dyadic Coping, and Overall Score increased gradually over time in both the experimental and control groups. However, the increase was greater in the experimental group. Pairwise comparisons using the Mann-Whitney U test showed similar outcomes: scores for these five dimensions and the overall score in the experimental group differed significantly from those in the control group at T2 and T3 (see Table 5).

## 4. Discussion

### 4.1. The Spousal Disclosure Intervention Scheme Constructed in This Study Demonstrates Good Compliance

This study applied an MSD to patients with gastric cancer and their spouses. In the experimental group, after eight weeks (T2), one patient was lost due to death. After 16 weeks (T3), one patient was lost due to the death of their spouse, and two patients were lost due to transfer to another hospital. This resulted in 38 couples completing the four sessions of MSD intervention and the third survey; no patients withdrew voluntarily, indicating good compliance. Research [26] confirms that the most common external barriers affecting emotional disclosure among cancer patients are the lack of adequate responses from family, friends, and other social support resources. For married cancer patients, spouses are usually their primary caregivers. Therefore, including patients’ spouses in the study and implementing a spousal self-disclosure intervention focusing on both patients and their spouses can improve patients’ compliance with emotional disclosure. Self-disclosure intervention, as a positive cognitive process therapy, can help patients reevaluate and adapt to stressful events, thereby alleviating their psychological distress [27]. The intervention in this study is divided into four phases: Personal Emotional Expression, Social Cognition Expression, Benefit Discovery, and Outlook to the Future, aimed at facilitating patients’ adaptation and self-regulation. Before implementing each phase, two experienced oncology nurses (Haiyan Zhao and Yuzhu Hou) provide MSD training for both the speaker and listener, enabling patients and their spouses to understand how to disclose appropriately. Through the progressive implementation of the four phases, disclosure skills are continuously reinforced, promoting mutual disclosure between spouses and enhancing marital intimacy. Meanwhile, two experienced oncology nurses (Haiyan Zhao and Yuzhu Hou) actively provide positive feedback and guidance on issues patients encounter during disclosure, further boosting patients’ and their spouses’ enthusiasm for participation and increasing the feasibility of the intervention scheme.

### 4.2. MSD Has a Significant Effect on Alleviating the FCR in Both Patients with Gastric Cancer and Their Spouses

The experimental group showed improved FCR levels after implementing MSD, with average scores of 27.05 and 23.16 at T2 and T3, respectively. In contrast, the control group had average scores of 30.86 and 30.03 at T2 and T3. A comparison between the two groups using Generalized Estimating Equations (GEE) showed that both groups improved FCR over time. However, the experimental group showed more significant improvement. The experimental group encouraged mutual disclosure between spouses by assessing couples’ existing communication patterns, providing training in communication skills and responsive listening, and training both spouses to express their inner thoughts verbally and in writing. When patients disclosed, their spouses responded supportively, helping them feel understood, satisfying their need to express themselves, and gaining their spouses’ support. This increased patients’ self-disclosure and reduced FCR for both patients and spouses, consistent with the results of Zhou et al. [13]. This study guided spouses in expressing concerns about cancer recurrence or progression, identifying patients’ specific fears, providing counseling, correcting misconceptions, evoking positive emotions, identifying beneficial aspects, and helping patients envision and plan for the future.

### 4.3. MSD Intervention Has a Positive Effect on Increasing the Dyadic Coping Ability of Couples

This study found that the between-group effects, time effects, and group-by-time interaction effects for the five DCI dimensions and the overall score were statistically significant (*p* < 0.05), except for the interaction effect for the Common Dyadic Coping dimension, which was not statistically significant (*p* > 0.05). These findings indicate that both groups showed significant improvements in dyadic coping ability over time, and significant between-group differences were observed for the DCI dimensions and overall score. For the Common Dyadic Coping dimension, although the group-by-time interaction effect was not statistically significant, significant between-group and time effects were observed, indicating that Common Dyadic Coping improved significantly over time and that the intervention group had a more favorable overall level than the control group. However, the scores for these five dimensions and the overall score in the experimental group differed significantly from those in the control group at T2 and T3 (*p* < 0.05). Self-disclosure is a form of communication in which individuals actively reveal their behaviors and inner emotions to others. The content of disclosure ranges from personal events to highly private, sensitive information. Research [28] shows that positive dyadic coping strategies between patients with chronic illness and their spouses not only promote patients’ physical and mental health and enhance quality of life but also improve relationship satisfaction and reduce spouses’ perceived caregiving stress. Only when partners understand the genuine emotions experienced by the other due to stress can they provide appropriately supportive dyadic coping that matches the genuine needs [29]. Similar to the findings of Wang et al. [30], self-disclosure intervention can promote the benefits of discovery for cancer patients, improve patients’ quality of life and psychological well-being, enhance communication and positive coping between couples, reduce negative emotions of patients and caregiving stress of partners, and strengthen the mutual coping ability of patient-couple pairs. The MSD program developed in this study trained couples in communication skills. It specified four phases of disclosure-focused work, helping them engage in reasonable stress release, enhancing the coping abilities of patients and partners, and improving the quality of life for both members of the couple.

Dyadic positive responses include positive communication, supportive binary responses, entrusted responses, and common binary responses, which reflect strong trust and a sense of belonging; negative coping manifests as reluctance, non-involvement, hostility, and protective buffering, indicating a lack of empathy and marital discord. The results also indicate that the experimental group showed higher levels of communication, supportive dyadic coping, delegated dyadic coping, and common dyadic coping than the control group (*p* < 0.05) after the MSD intervention. In contrast, the degree of Negative dyadic coping was lower than that in the control group (*p* < 0.05), indicating that MSD intervention can enhance positive coping abilities in stress, emotion, cooperation, and delegation for patients with gastric cancer and their spouses and reduce their negative coping. Self-disclosure between spouses can enhance intimacy and, in turn, improve their dyadic coping ability. Patients tend to be silent and anxious after being diagnosed, adopting avoidance and passive attitudes, which can lead to ineffective coping. Facilitating communication between spouses, encouraging patients to be open with their partners, and strengthening emotional support between spouses can elevate individual dual coping levels, consistent with the results of Ştefǎnuţ et al. [31]. Effective communication methods can increase marital intimacy, alleviate the pain caused by illness, boost patients’ confidence in coping with the disease, and promote supportive coping levels between both couples under disease-related stress [9]. Zhou et al. [26] suggest that patient self-disclosure intervention training, enhancing communication skills, promoting emotional expression between both parties, and analyzing innermost thoughts and feelings can deepen marital emotional relationships and strengthen collaborative dual coping abilities. The reasons behind this are, on the one hand, some patients with gastric cancer are willing to share their physical and psychological changes with their partners, forming a positive and effective communication pattern and enhancing patients’ positive coping mindset and abilities; on the other hand, partners, while accompanying patients, through correct disclosure methods, understand the support needed by patients in coping with the disease and provide targeted assistance. Both parties can adjust their coping methods promptly during communication to adapt to the disease’s dynamic changes.

### 4.4. Strengths of This Study

The MSD intervention targets emotional expression, social-cognitive sharing, benefit finding, and future outlook, and has been shown to reduce FCR compared with standard care. Early and continuous support is emphasized, as greater family support during chemotherapy is associated with lower FCR. Couples are trained in MSD techniques before formal disclosure, with review and practice at each phase, combining written and verbal methods to facilitate open discussion of FCR concerns. This intervention, led by two experienced oncology nurses (Haiyan Zhao and Yuzhu Hou), also teaches principles of respect, empathy, and positive attention, helping nurses build supportive, trusting relationships while enhancing marital communication and psychological adjustment.

### 4.5. Limitations of This Study

Although the two participating hospitals belonged to the same medical alliance, were both tertiary-level hospitals, and provided comparable routine nursing care, institutional-level differences in healthcare resources, clinical practice patterns, and patient characteristics could not be entirely ruled out, as only two hospitals were included, with one assigned to each study group. Baseline characteristics of enrolled participants showed no significant between-group differences, and both hospitals maintained usual care during the study period. Furthermore, the control hospital provided no additional psychological or structured psychosocial interventions to patients or their spouses during the study. Nevertheless, these measures cannot fully eliminate potential institutional-level selection bias. Future multicenter studies involving more hospitals and cluster-level randomization are warranted to improve the internal validity and generalizability of the findings.

Most couples in this study had been married for over 20 years, and most patients were male, which may have introduced bias. In addition, the study was conducted within a specific cultural and healthcare context in China; therefore, the findings may not generalize directly to couples in other cultural settings or healthcare systems, where marital communication, self-disclosure, and access to psychosocial care may differ. Future cross-cultural and multicenter studies are needed to examine MSD applicability across different populations and healthcare contexts. The relatively short, 16-week follow-up also limited the assessment of whether improvements in FCR and dyadic coping could be sustained over time. Longer-term follow-up is therefore recommended to evaluate the durability of the intervention effects.

Since cancer impacts not only patients and spouses but also the entire family, changes in family communication and roles may affect marital interactions, suggesting the need for further research on family communication. With the growth of mobile health and 5G technology, online psychotherapy offers flexible social and psychological support for patients and spouses. Future studies could investigate the effectiveness of online MSD interventions. Additionally, integrating MSD into routine nursing practice to improve efficiency remains a practical consideration that warrants further exploration.

### 4.6. Implications of the Study

Oncology nurse-led psychological interventions are a key element of supportive care for cancer patients. According to the UK National Institute for Health and Care Excellence (NICE) guidelines, these interventions should be central to psychological support. Oncology nurses can assess patients’ psychological status and deliver evidence-based care for identified psychological concerns. Patients often prefer nurse-led support during treatment, and such interventions are generally well accepted by those experiencing psychological distress.

Clinical implications. The MSD intervention was specifically designed for delivery by oncology nurses and was further refined through consultation with experts in the field. The intervention development process was rigorous and scientifically grounded, and patients demonstrated good adherence throughout. The significant improvements in FCR among both patients and their spouses, as well as in couples’ dyadic coping, indicate that nurse-led MSD may be an effective and feasible approach for addressing psychological and relational needs during chemotherapy. Clinically, oncology nurses may incorporate structured couple-based self-disclosure into routine supportive care to facilitate communication between patients and spouses, strengthen dyadic coping, and reduce FCR. The intervention may also provide a practical framework for oncology nurses to identify and address both individual and couple-level psychosocial needs during cancer treatment.

Research implications. This study provides preliminary evidence for the potential value of nurse-led MSD in improving psychological and dyadic outcomes among couples coping with cancer. Future studies could examine the effectiveness of MSD in larger and more diverse samples, as well as across different cancer populations and treatment settings. Further research is also needed to determine the intervention’s long-term effects, identify the mechanisms underlying its effects on FCR and dyadic coping, and explore its feasibility and cost-effectiveness in routine oncology practice. These studies could further refine and broaden the implementation of nurse-led, couple-based psychological interventions.

## 5. Conclusions

The MSD intervention was developed through a scientific and feasible process, with good adherence among patients with gastric cancer and their spouses. After 8 and 16 weeks of intervention, MSD significantly reduced FCR in both patients and spouses and improved couples’ dyadic coping by reducing negative dyadic coping and enhancing supportive dyadic coping, stress communication, delegated dyadic coping, and common dyadic coping. These findings suggest that MSD may be a promising nurse-led, couple-based psychological intervention for addressing FCR and strengthening couples’ coping with cancer. The study also highlights the importance of involving both patients and spouses in oncology nursing care and provides a potential approach for integrating dyadic psychological support into clinical practice. Future research should further validate the effectiveness and long-term effects of MSD through larger multicenter studies and explore its applicability to other cancer populations and cultural contexts.

## Figures and Tables

**Table 1:** General Information and Comparison of Patients with Gastric Cancer in Two Groups (*n* = 84).

Characteristics		Total Number (*n*)	Experimental Group (*n*)	Control Group (*n*)	*χ*^2^/Fisher	*p*
Gender	Male	63	32	31	0.063	0.801 ^a^
	Female	21	10	11		
Age	20–30	1	1	0	Fisher = 5.471	0.304 ^b^
	31–40	1	0	1		
	41–50	6	1	5		
	51–60	22	12	10		
	61–70	34	16	18		
	Over 70	20	12	8		
Duration of Marriage (years)	Less than 10	1	1	0	Fisher = 4.346	0.184 ^b^
	10–20	3	0	3		
	21–30	31	14	17		
	Over 30	49	27	22		
Number of Children	None	6	5	1	Fisher = 4.279	0.214 ^b^
	One child	48	24	24		
	Two children	28	13	15		
	More than Two	2	0	2		
Resident	Urban	28	12	16	1.333	0.513 ^a^
	Suburban	21	10	11		
	Rural	35	20	15		
Religious Belief	None	83	41	42	-	1.000 ^b^
	Yes	1	1	0		
Health Insurance Type	Basic Social Medical Insurance	83	41	42	-	1.000 ^b^
	Commercial Insurance	0	0	0		
	Self-paid	1	1	0		
	Other	0	0	0		
Medical Burden	Completely burden-free	5	4	1	5.011	0.164 ^a^
	Basic burden-free	25	12	13		
	Some burden	45	24	21		
	Heavy burden	9	2	7		
Average Monthly Household Income per Capita (RMB)	Less than 2000	26	17	9	Fisher = 7.976	0.115 ^b^
	2001–4000	20	7	13		
	4001–6000	28	11	17		
	6001–8000	6	4	2		
	8001–10,000	2	2	0		
	Over 10,001	2	1	1		
Educational Level	Primary school and below	31	14	17	Fisher = 3.856	0.388 ^b^
	Junior high school	40	23	17		
	High school or vocational school	11	4	7		
	Bachelor’s degree or college	1	1	0		
	Master’s or above	1	0	1		
Employment Status	Employed	11	6	5	1.091	0.580 ^a^
	Unemployed	73	36	37		
Current or former occupation	Have not worked	32	19	13	Fisher = 3.036	0.785 ^b^
	Worker, Farmer	33	15	18		
	Business, Service work	2	1	1		
	Teacher	1	0	1		
	Medical worker	0	0	0		
	Civil servants and staff of public institutions	4	2	2		
	Other	12	5	7		
History of Previous Gastric Surgery	No	39	18	21	1.322	0.516
	Yes	45	24	21		
Cancer Stage	Stage I	0	0	0	Fisher = 0.192	1.000 ^b^
	Stage II	11	5	6		
	Stage III	48	24	24		
	Stage IV	25	13	12		
Chemotherapy regimen	Epirubicin + Cisplatin + Fluorouracil	3	1	2	4.585	0.469 ^a^
	Docetaxel + Cisplatin + Fluorouracil	3	1	2		
	Epirubicin+ Oxaliplatin + Capecitabine	8	6	2		
	Oxaliplatin+ Capecitabine or Docetaxel + Leucovorin + Fluorouracil + Oxaliplatin	47	25	22		
	Other	23	9	14		
Time of Cancer Diagnosis (months)	Less than 1	4	1	3	2.068	0.558 ^a^
	1–<2	30	15	15		
	2–3	18	11	7		
	Over 3	32	15	17		
Your Understanding of Your Condition	Completely understood	44	24	20	-	0.512 ^b^
	Partially understood	40	18	22		
	Misunderstand	0	0	0		

Note: ^a^ = Chi-Square χ^2^; ^b^ = Fisher’s exact value.

**Table 2:** General Information and Comparison of the Spouses of Patients with Gastric Cancer in Two Groups (*n* = 84).

Characteristics		Total Number (*n*)	Experimental Group (*n*)	Control Group (*n*)	*χ*^2^/Fisher	*p*
Age	20–30	0	0	0	Fisher = 7.416	0.094 ^b^
	31–40	1	1	0		
	41–50	12	2	10		
	51–60	17	10	7		
	61–70	39	20	19		
	>70	15	9	6		
Education Level	Primary school or below	43	22	21	Fisher = 1.716	0.939 ^b^
	Junior high school	30	16	14		
	Senior high school or vocational school	7	3	4		
	College or junior college	3	1	2		
	Bachelor’s degree or above	1	0	1		
Occupation	Unemployed	28	18	10	Fisher = 6.077	0.264 ^b^
	Worker, farmer	22	10	12		
	Business, Service work	18	9	9		
	Teacher	2	0	2		
	Medical worker	0	0	0		
	Civil servants or staff of public institutions	2	0	2		
	Other	12	5	7		
How do you feel about your spouse’s understanding of your illness	Fully understand	4	3	1	3.013	0.222
	Partially understand	35	14	21		
	misunderstand	45	25	20		

Note: ^b^ = Fisher’s exact value.

**Table 3:** Comparison of FoP-Q-SF for Patients and FoP-Q-SF for Partners in Two Groups (Intention-to-Treat Analysis).

Group	Case	FoP-Q-SF for Patients (Score)			Wald χ^2^_group_/ *p*-Value	Wald χ^2^_time_/ *p*-Value	Wald χ^2^_Group×Time_/ *p*-Value
		T1	T2	T3			
Experimental Group	42	36.5 (31–38)	26.5 (23–30)	24 (18.44–27)	10.124/0.001	128.786/<0.001	36.638/<0.001
Control Group	42	33 (30–37)	30 (26.21–33)	29.79 (25–34)			
Z-value		−1.901	−2.728	−4.551			
*p*-value		0.057	0.006	<0.001			
Experimental Group	42	34.5 (31–38)	27.5 (24–30)	27.5 (24–30)	10.408/0.001	63.928/<0.001	18.309/<0.001
Control Group	42	35 (30–38)	30.62 (27.75–35)	30.62 (27.75–35)			
Z-value		−0.409	−3.445	−4.138			
*p*-value		0.683	0.001	<0.001			

Note: Data are median [IQR (Q25–Q75)], T1 (Baseline), T2 (8 weeks after intervention), and T3 (16 weeks after intervention).

**Table 4:** The Scores of DCI in the Two Groups.

Scale Dimension	Number of Items (Score Range)	Experimental Group (Score)			Control Group (Score)		
		T1 (*n* = 42)	T2 (*n* = 41)	T3 (*n* = 38)	T1 (*n* = 42)	T2 (*n* = 36)	T3 (*n* = 36)
Negative dyadic coping	8 (8~40)	29 (27–31) ^a^	32 (31–38) ^a^	34.32 (4.48) ^b^	25.5 (20–34.5) ^a^	29 (20–34.5) ^a^	28.08 (7.62) ^b^
Stress communicate	8 (8~40)	26.03 (4.86) ^b^	29 (26–32) ^a^	32 (30–32) ^a^	25.75 (4.48) ^b^	28 (23–31) ^a^	26.64 (5.13) ^b^
Supportive dyadic coping	10 (10~50)	32.05 (5.76) ^b^	38 (35–40) ^a^	40 (38–40) ^a^	31.92 (6.12) ^b^	34 (27–37) ^a^	35 (29–37) ^a^
Delegated dyadic coping	4 (4~20)	13.5 (12–15) ^a^	15 (14–16) ^a^	16 (15–16) ^a^	13.31 (2.56) ^b^	14 (12.5–16) ^a^	14 (12.5–15) ^a^
Common dyadic coping	5 (5~25)	19 (17–20) ^a^	20 (19–20) ^a^	20 (20–21) ^a^	16.56 (3.84) ^b^	18.5 (14.5–20) ^a^	19 (14.5–20) ^a^
Overall score	35 (35~175)	119.5 (113–127) ^a^	132.45 (14.82) ^b^	140.29 (11.80) ^b^	114.75 (19.75) ^b^	118.39 (20.73) ^b^	118.97 (20.59) ^b^

Note: ^a^ = Median [IQR (Q25–Q75)], ^b^ = Mean (SD). T1 (baseline), T2 (8 weeks after intervention), and T3 (16 weeks after intervention).

**Table 5:** Comparison of DCI in Two Groups (Intention-to-Treat Analysis).

Group	Case	Negative Dyadic Coping (Score)			Wald χ^2^_group_/ *p*-Value	Wald χ^2^_time_/ *p*-Value	Wald χ^2^_Group×Time_/ *p*-Value
		T1 (*n* = 42)	T2 (*n* = 42)	T3 (*n* = 42)			
Experimental Group	42	29 (28–31)	32.5 (32–38)	33.5 (32–39)	9.162/0.002	67.104/<0.001	29.066/<0.001
Control Group	42	29.5 (20–35)	31 (20–35.25)	31 (22–35.91)			
Z-value		−0.153	−2.843	−3.408			
*p*-value		0.879	0.004	0.001			
Group	Case	Stress communicate (score)			Wald χ^2^_group_/*p*-value	Wald χ^2^_time_/*p*-value	Wald χ^2^_group×time_/ *p*-value
		T1 (*n* = 42)	T2 (*n* = 42)	T3 (*n* = 42)			
Experimental Group	42	26 (24–30)	32.5 (32–38)	32 (30–32)	8.505/0.004	41.054/<0.001	11.369/0.003
Control Group	42	25 (23–29)	31 (20–35.25)	28 (24–31)			
Z-value		−0.768	−2.228	−4.094			
*p*-value		0.443	0.026	<0.001			
Group	Case	Supportive dyadic coping (score)			Wald χ^2^_group_/*p*-value	Wald χ^2^_time_/*p*-value	Wald χ^2^_group×time_/ *p*-value
		T1 (*n* = 42)	T2 (*n* = 42)	T3 (*n* = 42)			
Experimental Group	42	33 (30–36)	38 (35–40)	40 (38–40)	11.109/0.001	56.029/<0.001	16.651/<0.001
Control Group	42	31 (28–36)	34 (27.28–37)	35 (29.93–37)			
Z-value		−1.009	−3.068	−4.602			
*p*-value		0.313	0.002	<0.001			
Group	Case	Delegated dyadic coping (score)			Wald χ^2^_group_/*p*-value	Wald χ^2^_time_/*p*-value	Wald χ^2^_group×time_/ *p*-value
		T1 (*n* = 42)	T2 (*n* = 42)	T3 (*n* = 42)			
Experimental Group	42	13 (12–15)	15 (14–16)	16 (15–16)	4.102/0.043	46.631/<0.001	20.484/<0.001
Control Group	42	13 (12–16)	14 (13–16)	14 (13–15.89)			
Z-value		−0.583	−2.601	−4.027			
*p*-value		0.560	0.009	<0.001			
Group	Case	Common dyadic coping (score)			Wald χ^2^_group_/*p*-value	Wald χ^2^_time_/*p*-value	Wald χ^2^_group×time_/ *p*-value
		T1 (*n* = 42)	T2 (*n* = 42)	T3 (*n* = 42)			
Experimental Group	42	19 (17–20)	20 (19–20)	20 (20–21)	7.345/0.007	30.952/<0.001	3.720/0.156
Control Group	42	17 (15–20)	18.08 (15–20)	19 (15–20)			
Z-value		−1.874	−2.33	−3.534			
*p*-value		0.061	0.020	<0.001			
Group	Case	Overall score (score)			Wald χ^2^_group_/*p*-value	Wald χ^2^_time_/*p*-value	Wald χ^2^_group×time_/ *p*-value
		T1 (*n* = 42)	T2 (*n* = 42)	T3 (*n* = 42)			
Experimental Group	42	119.5 (113–127)	136 (128–142)	140 (133.16–148)	13.025/<0.001	100.233/<0.001	30.124/<0.001
Control Group	42	113.5 (103–127)	119.5 (109–132)	121.16 (109–139.07)			
Z-value		−1.379	−3.45	−4.649			
*p*-value		0.168	0.001	<0.001			

Note: Data are Median [IQR (Q25–Q75)]. T1 (Baseline), T2 (8 weeks after intervention), and T3 (16 weeks after intervention).

## Data Availability

The data presented in this study are available upon request from the corresponding author for ethical reasons.
